# The Median Nerve Injury Model in Pre-clinical Research – A Critical Review on Benefits and Limitations

**DOI:** 10.3389/fncel.2019.00288

**Published:** 2019-06-28

**Authors:** Giulia Ronchi, Michela Morano, Federica Fregnan, Pierfrancesco Pugliese, Alessandro Crosio, Pierluigi Tos, Stefano Geuna, Kirsten Haastert-Talini, Giovanna Gambarotta

**Affiliations:** ^1^Department of Clinical and Biological Sciences, University of Turin, Turin, Italy; ^2^Neuroscience Institute Cavalieri Ottolenghi Foundation (NICO), University of Turin, Turin, Italy; ^3^Dipartimento di Chirurgia Generale e Specialistica, Azienda Ospedaliera Universitaria, Ancona, Italy; ^4^UO Microchirurgia e Chirurgia della Mano, Ospedale Gaetano Pini, Milan, Italy; ^5^Institute of Neuroanatomy and Cell Biology, Hannover Medical School, Hanover, Germany; ^6^Center for Systems Neuroscience (ZSN) Hannover, Hanover, Germany

**Keywords:** median nerve, injury, animal experimental model, repair, regeneration, translational research

## Abstract

The successful introduction of innovative treatment strategies into clinical practise strongly depends on the availability of effective experimental models and their reliable pre-clinical assessment. Considering pre-clinical research for peripheral nerve repair and reconstruction, the far most used nerve regeneration model in the last decades is the sciatic nerve injury and repair model. More recently, the use of the median nerve injury and repair model has gained increasing attention due to some significant advantages it provides compared to sciatic nerve injury. Outstanding advantages are the availability of reliable behavioural tests for assessing posttraumatic voluntary motor recovery and a much lower impact on the animal wellbeing. In this article, the potential application of the median nerve injury and repair model in pre-clinical research is reviewed. In addition, we provide a synthetic overview of a variety of methods that can be applied in this model for nerve regeneration assessment. This article is aimed at helping researchers in adequately adopting this *in vivo* model for pre-clinical evaluation of peripheral nerve reconstruction as well as for interpreting the results in a translational perspective.

## Introduction

Peripheral nerve injuries are commonly caused by motor vehicle, domestic, work or sport accidents or during surgeries (iatrogenic nerve injuries) (Jones et al., 2016). Nerve injuries can lead to motor and sensory deficits that may result in disabilities permanently compromising the patients’ quality of life.

The general ability of peripheral nerves to regenerate has been recognised more than a century ago, but until today functional recovery outcome after severe nerve injury and reconstructive surgery is often still poor in many patients. Nowadays, the “gold standard” reconstructive technique for bridging a nerve gap is autologous nerve grafting. This technique, however, is accompanied by important drawbacks such as the donor site morbidity, the need of additional surgery and the limited availability of graft material for extended repair (Konofaos and Ver Halen, 2013; Faroni et al., 2015).

Over the past decades, substantial effort has been made to identify new strategies to improve peripheral nerve regeneration after grafting and to substitute the autologous nerve graft. Advancements in biomedical methods, the tissue-engineered technology, gene therapy approaches, nanotechnology, biology, and microsurgical skills have opened new research fields in the nerve reconstruction area. Indeed, there is an exponential increase in the number of publications dealing with experimental nerve regeneration research over the years: a literature search with the PubMed search string (“Nerve-Regeneration”[Mesh] OR nerve-regenerat^∗^ OR nerve-repair^∗^) AND (rat^∗^ OR mouse OR mice OR rabbit^∗^ OR sheep), delivered 26 results in 1970 and 479 in 2018.

In the context of pre-clinical peripheral nerve regeneration research, the choice of the experimental animal model is of fundamental importance. When a researcher moves on to test a novel attempt *in vivo*, the animal model should be chosen according to the study aims [e.g., for studying the involvement of a specific molecule in the biological process of nerve regeneration, the most appropriate choice will most likely be different to the model chosen for evaluating the effectiveness of a nerve conduit for long gap (>50 mm) repair]. Obviously, the pros and cons of the different available options must also be taken into careful consideration.

The choice of the appropriate experimental nerve injury model is usually guided by several factors. For nerve repair studies, in particular, the size (diameter and length) of the model nerve is certainly one of the main aspects considered. Indeed, most nerve repair studies are conducted on the sciatic nerve especially because of its big dimension that facilitates experimental microsurgery (Varejao et al., 2004; Bozkurt et al., 2011; Sinis et al., 2011a). The sciatic nerve is the biggest nerve in the body and the choice among mouse, rat, rabbit, dog, or sheep already provides variability in nerve gap lengths to be applied (Angius et al., 2012).

Different downsides resulting from experimental injury to the sciatic nerve have, however, led to increasing interest in the median nerve as alternative model nerve (Bertelli et al., 2004). At first, injury to the sciatic nerve results in a paralysis of the hind limb and, often, in automutilation behaviour, such as biting and self-amputation of denervated toes and paw areas by the subjected animal. Longer lasting paralysis (>4 weeks in the rat) often leads to joint contractures and stiffness. Automutilation behaviour and joint contractures reduce the reliability of functional tests, such as estimation of the sciatic function index or, in severe cases, will lead to exclusion of the respective animal from a study for ethical and animal welfare reasons. Furthermore, possibilities for evaluation functional recovery of motor skills after sciatic nerve lesion in the awaken animal are rather limited or need considerable efforts to be realised (Navarro, 2016).

In the recent years employment of the median nerve injury and repair model in the experimental research has increased (Papalia et al., 2003b; Ronchi et al., 2009) because of several advantages. Transection injury of the rodent median nerve, results in only partial impairment of the upper limb function (Bertelli et al., 1995). Incidence of automutilation is significantly lower in comparison to the sciatic nerve model, ulcerations are fewer and no joint contractures can be seen. This milder phenotype results from the fact that after median nerve injury, the ulnar and radial nerves still preserve sensitivity and motor function in the forearm (Sinis et al., 2006). An additional advantage of the rodent median nerve injury and repair model is that positively evaluated attempts are more likely to be translated into clinical practise, since surgical interventions for the repair of a damaged human nerve are very often performed at the upper limb level. In addition, the hand functions require a fine finger movement that is quite similar between rodents and humans (Whishaw et al., 1992). From this perspective, the possibility to apply specific and precise functional tests for motor recovery evaluation following median nerve reconstruction is a further pro of this model in pre-clinical research.

This review provides an overview on the use of the median nerve injury and repair experimental model in pre-clinical research. The different animal species (not only mouse and rat but also larger animals such as rabbit, sheep, and monkey) are taken into account as are the different options these species provide with regard to comprehensive analysis of the regeneration outcome.

## The Pre-Clinical Median Nerve Injury and Repair Model in Different Animal Species

The use of the median nerve injury and repair model as pre-clinical model has progressively increased in the last years, but the sciatic nerve injury and repair model is still more often employed [in PubMed a research on (median-nerv^∗^) AND (regenerat^∗^ OR repair) yielded 1002 papers, while (sciatic-nerv^∗^) yielded 6612 papers].

Information from our literature search is presented in Tables 1–5. The tables summarise peripheral nerve regeneration studies after median nerve injury and repair in the different animal models. In addition to the specific reference, the tables list animal strain and sex, type of injury/gap, follow up periods and type of analyses conducted. We took our best efforts to include all articles available until end of 2018; nevertheless, inadvertently, we could have missed some papers and apologise in advance with their authors.

**Table 1:** Mouse model.

References	Strain	Sex	Type of injury/gap	Follow up	Analysis
Jager et al., 2014	–	F	Crush injury (*n* = 5); Contralateral nerves used as control (uninjured) nerves	25 days	Functional analysis (grasping test), histological analysis, TEM, morphometry
Park and Hoke, 2014	C57Bl/6J	M	Nerve repair (microsurgical 10/0 suture) without Exercise (*n* = 8); Nerve repair (microsurgical 10/0 suture) with Exercise (*n* = 8); Control group (*n* = 8)	6 weeks	Functional test, Electrophysiology, morphometry, Immunohistochemistry, ELISA assay (serum sample)
Speck et al., 2014	Swiss mice	M	Crush injury (*n* = 12); Control group (*n* = 12)	21 days	Functional test (IBB – Irvine, Beatties, and Bresnahan – Forelimb Scale), Histology
Jaminet et al., 2013b	CD1 and C57BL/6	M	Immediate microsurgical repair using 12/0 sutures (*n* = 48 WT); Immediate microsurgical repair using 12/0 sutures (*n* = 8 WT); Control (*n* = 8 WT) Immediate microsurgical repair using 12/0 sutures (*n* = 8 heterozygous Netrin-1(+/-); Control (*n* = 8 heterozygous Netrin-1(+/-)	0, 7, 14, 21, and 50 days	Real-time PCR, Western Blot, TEM, morphometry, functional analysis (grasping test)
Jaminet et al., 2013a	C57BL/6	–	Immediate microsurgical repair using 12/0 sutures (*n* = 24 WT); Immediate microsurgical repair using 12/0 sutures (*n* = 12 WT); Control (*n* = 12 WT) Immediate microsurgical repair using 12/0 sutures (*n* = 12 UNC5b^+/-^ heterozygous); Control (*n* = 12 UNC5b^+/-^ heterozygous)	0, 7, 14, 21, and 50 days	Western Blot, TEM, morphometry, functional analysis (grasping test)
Ronchi et al., 2013	BALB/c	M	Crush injury (*n* = 16 BALB-neuT); Crush injury (*n* = 16 WT); Contralateral nerves used as control (uninjured) nerves	2 and 28 days	Functional analysis, immunohistochemistry, histology, stereological analyses, TEM, western blot, real-time PCR
Oliveira et al., 2010	C57/Black6	–	Nerve lesion followed by tubulization [polycaprolactone (PCL) conduits] with DMEM (*n* = 10), 3-mm gap; Nerve lesion followed by tubulization (PCL conduits) with MSC in DMEM (*n* = 11), 3-mm gap Control (*n* = 10)	4, 8, and 12 weeks	SEM, TEM, histomorphometry analysis, immunohistochemistry, functional analysis
Ronchi et al., 2010	FVB	M	Crush injury (5 animals); Microsurgical 12/0 suture (end-to-end neurorrhaphy) (*n* = 6) and controls (*n* = 6) from Tos et al. (2008)	25 days	Functional analysis, histology, stereological analyses
Tos et al., 2008	FVB	M	Microsurgical 12/0 suture (end-to-end neurorrhaphy) (*n* = 6); Controls (*n* = 6)	75 days	Functional, histology, stereological analyses, TEM

**Table 2:** Rat model.

References	Strain	Sex	Type of injury/gap	Follow up	Analysis
Casal et al., 2018	Wistar	F	Control (*n* = 17) Excision (*n* = 17) 10 mm autograft (*n* = 19); Conventional flap (*n* = 19); Arterialised venous nerve flap (*n* = 15); Prefabricate nerve flap (*n* = 8)	100 days	Grasping test; nociception evaluation; running velocity; walking track analysis, retrograde labelling, infra-red thermography, electroneuromyography, immunohistochemistry
Chen et al., 2018	Sprague-Dawley	M	Control (*n* = 6); Nerve constriction with four loose ligatures for 1 (*n* = 6), 2 (*n* = 4), 3 (*n* = 4), and 4 weeks (*n* = 4); Median nerve transection (*n* = 5), 1 week intraplantar administration of saline, M871 (a GalR2 antagonist), or AR-M1896 (a GalR2 agonist)	1, 2, 3, and 4 weeks	Immunocytochemistry, von Frey filaments test
Gao et al., 2018	Sprague-Dawley	F	Entire contralateral C7 root was transected and transferred to the median nerve (*n* = 18); Only the posterior division of the contralateral C7 root was transected and transferred to the median nerve (*n* = 18); The entire contralateral C7 root was transected but only the posterior division was transferred to the median nerve (*n* = 18)	8, 12, and 16 weeks	Electrophysiological examination, Muscle tetanic contraction force test, Muscle fibre cross-sectional area, histological and morphometrical analysis
Gluck et al., 2018	Sprague-Dawley	F	Control (*n* = 10); Low strain injury (strain of 14% elongation) (*n* = 5/time point); High strain injury (strain of 20% elongation) (*n* = 5/time point)	0, 1, 3, 8, and 12 weeks	Second Harmonic Generation (SHG) microscopy, histology, and Immunohistochemistry
Marchesini et al., 2018	Wistar	M	Autograft – 1.5 cm gap (*n* = 6) 1.5 cm median nerve gap repaired with amnion muscle combined graft (AMCG) conduits (*n* = 8)	90 days	Grasping test, histological and morphometrical analysis
Marcioli et al., 2018	Wistar	M	Neural compression without treatment (*n* = 6); Neural compression and treated with neural mobilisation for 1 min (*n* = 6); then 6 sessions on alternate days of mobilisation; Neural compression and treated with neural mobilisation for 3 min (*n* = 6); then 6 sessions on alternate days of mobilisation	14 days	Histology and morphometric analysis, PCR
Muratori et al., 2018	Wistar	F	Control (*n* = 6); Crush injury (*n* = 3/time point + 3 for histology); End-to-end repair (*n* = 3/time point); Degenerating nerve (*n* = 3/time point)	1, 3, 7, 15, and 30 days	Real time PCR, western blot, immunohistochemistry
Ronchi et al., 2018	Wistar	F	Nerve repaired with chitosan conduit (10 mm long) (*n* = 5 for each time point); Nerve repaired with chitosan conduit (10 mm long) filled with fresh muscle fibres (*n* = 5 for each time point); Autograft (*n* = 5 for each time point)	1, 7, 14, and 28 days and 12 weeks	Grasping test, histological analysis, morphometrical analysis,
Meyers et al., 2017	Sprague-Dawley	F	End-to-end repair of the median nerve distal to the elbow (*n* = 6); End-to-end repair of the median and ulnar nerves proximal to the elbow (*n* = 6); Repair of the median and ulnar nerves with a 7-mm polyurethane tube (gap 5 mm) proximal to the elbow (*n* = 9)	Up to 13 weeks	Evaluation of volitional forelimb strength
Ronchi et al., 2017	Wistar	F	Immediate, 3 and 6 months delayed nerve repair with a cross suture between the degenerated median nerve distal stump and the freshly axotomised ulnar proximal stump (*n* = 7/group); Controls: healthy nerve (*n* = 5); 9-month degenerated nerve (*n* = 5); 3-month regenerated end-to-end–repaired median nerves (*n* = 5)	6 months	Grasping test, histological analysis, morphometrical analysis, gene expression analysis, protein analysis
Stossel et al., 2017	Lewis	F	7 mm-long nerve repaired with muscle-in vein graft (*n* = 8); Control: autologous nerve graft (*n* = 8)	1, 2, and 3 months	Electrophysiology, grasping test, staircase test, histological analysis, morphometrical analysis
Coradini et al., 2015	Wistar	M	Healthy nerve in obese rat model (8 rats); Crush nerve injury in obese rat model (8 rats); Crush nerve injury and physical exercise (swimming) in obese rat model (8 rats); Controls: healthy nerve (8 rats); crush nerve injury (8 rats); crush nerve injury plus physical exercise (8 rats)	3, 7, 14, and 21 days	Nociception threshold, histological analysis, protein analysis
Fregnan et al., 2016	Wistar	F	10 mm-nerve gap repaired with two different types of chitosan membrane based conduit (*n* = 4/group); Control: autograft (*n* = 4)	3 months	Grasping test, histological analysis, morphometrical analysis
Huang and Tsai, 2016	Sprague–Dawley	M	Control (*n* = 12); Median nerve compression with four loose ligatures (*n* = 12/time point) with delivery of the JNK inhibitor at different doses [20 (*n* = 6), 40 (*n* = 6), or 80 nmol (*n* = 6), or with vehicle (*n* = 6)]; Animals were given vehicle (*n* = 18) or DHA at doses of 100 (*n* = 18), 250 (*n* = 18), or 500 nmol/kg (*n* = 18)	5 h, 1, 3, 5, 7, 14, and 21 days	Immunohistochemistry, double-immunofluorescence labelling and Western blotting, Enzyme-linked immunosorbent assay, Behavioural testing
Papalia et al., 2016	Wistar	F	10 mm-nerve gap repaired with end-to-side neurorrhaphy with or without epineurial window, using ulnar nerve fixed by applying cyanoacrylate solution (*n* = 7/group); Control: healthy nerve (*n* = 5)	9 months	Grasping test, histological analysis, morphometrical analysis
Ronchi et al., 2016	Wistar	F	Crush nerve injury (*n* = 15); Nerve transection and immediately repair with end-to-end technique (*n* = 15); Nerve transection followed by no repair (*n* = 15); Control: healthy nerve (*n* = 7)	1, 3, 7, 14, and 28 days	Histological analysis, gene expression analysis, protein analysis
Shaikh et al., 2016	Long Evans	F	Nerve transection and nerve wrapping with a strip of Rose Bengal chitosan adhesive followed by laser irradiation (*n* = 10); Nerve transection repaired with end-to-end neurorrhaphy (*n* = 10); Control: nerve wrapping with a strip of Rose Bengal chitosan adhesive followed by laser irradiation (*n* = 10)	3 months	Cold and warm plate test, withdrawal threshold tests
Gambarotta et al., 2015	Wistar	F	Transected nerve repaired with 10 mm-long muscle-in-vein graft with muscle fibres expressing AAV2-LacZ or AAV2-ecto-ErbB4 (*n* = 5/group); Control: 10 mm-long nerve graft (*n* = 5)	3 months	Grasping test, histological analysis, morphometrical analysis
Beck-Broichsitter et al., 2014b	Wistar	F	Nerve transection repaired with direct coaptation plus pulsed magnetic therapy (*n* = 12); Controls: nerve transection repaired with direct coaptation (*n* = 12)	3 months	Grasping test, histological analysis, morphometrical analysis
Beck-Broichsitter et al., 2014a	Wistar	F	6 weeks delayed nerve repair with autograft (*n*=); Nerve injury with sensory protection and 6 weeks delayed repair with autograft (*n* = 10)	15 weeks	Grasping test, muscle weight, histological analysis, morphometrical analysis
Li et al., 2014	Sprague-Dawley	–	Acute group (immediately after injury, *n* = 18) and subacute (2 weeks after injury, *n* = 18) group, each divided in three subgroups: Nerve transected without repair; Nerve transected and repaired immediately; Healthy control	Immediately and 2 weeks after injury	fMRI/fcMRI
Manoli et al., 2014	Wistar	F	Direct suture (*n* = 3); Direct suture plus vein-graft wrapping (*n* = 4); Direct suture plus vein-graft wrapping filled with Perineurin vehicle (*n* = 2); Direct suture plus vein-graft wrapping filled with Perineurin (*n* = 6); Direct suture (*n* = 5) FloSeal application to the nerve stumps and direct suture (*n* = 6); Electrocoagulation of the nerve stumps and direct suture (*n* = 5)	12 weeks	Electrophysiological and histomorphological analysis
Oliveira et al., 2014	Wistar	M	Nerve transection (4 mm gap) repaired with polycaprolactone conduit, with an injection of cell medium alone (*n* = 3) or containing bone marrow-derived mesenchymal stem cell (*n* = 3); Control: healthy nerve (*n* = 4)	10 weeks	Histological analysis, morphometrical analysis, electrophysiological cortical mapping of the somatosensory representation
Ronchi et al., 2014	Wistar	F	Control (*n* = 5); Crush injury (*n* = 5); Autograft (*n* = 5)	12 weeks	Histological and morphometrical analysis (at light and electron microscopy)
Ghizoni et al., 2013	Sprague-Dawley	F	40 mm nerve gap repaired with autograft (contralateral median nerve) and nandrolone treatment (*n* = 20) 40 mm nerve gap repaired with autograft (contralateral median nerve) (*n* = 20); Control: non-grafted animals (*n* = 20)	6 months	Electrophysiology, grasping test, muscle weight, nociceptive sensation recovery
Ho et al., 2013	Sprague-Dawley		Nerve repaired with silicone rubber tubes (gap 5 mm) and subjected to acupuncture and electroacupuncture at two different intensities (*n* = 7/group); Control: nerve repaired with silicone rubber tubes (gap 5 mm); no stimulation	5 weeks	Electrophysiology, Histology, Grasping test
Marcioli et al., 2013	Wistar	M	Nerve compression and treatment with neural mobilisation for 1 or 3 min (*n* = 6/group); Control: nerve compression without mobilisation (*n* = 6)	3, 5, 7, 11, and 13 days, 2 weeks	Nociception evaluation, histological Analysis, morphometrical analysis
Moimas et al., 2013	Wistar	F	Transected nerve repaired using 10 mm-long muscle-in-vein graft with muscular fibres expressing AAV2-LacZ or AAV2-VEGF (*n* = 7/group). Control: healthy nerve (*n* = 5)	3 months	Grasping test, histological analysis and morphometrical analysis of both nerve and muscle, muscle immunochemistry
Papalia et al., 2013	Wistar	F	10 mm-long defect repaired with adipose tissue-in-vein conduit (*n* = 5) or muscle-in-vein conduit (*n* = 5); Control: autologous nerve graft (*n* = 5)	6 months	Grasping test, histological analysis, morphometrical analysis
Lanza et al., 2012	Wistar	F	Nerve transection immediately repaired with end-to-side neurorrhaphy (*n* = 10); 2 mm-long nerve segment exported and not repaired (*n* = 10)	6 and 12 days	Histological analysis, gene expression analysis
Muratori et al., 2012	Wistar	F	Crush injury (*n* = 5); Control: healthy nerve (*n* = 5)	8 and 24 weeks	Histological analysis, morphometrical analysis
Moges et al., 2011	Sprague Dawley	F	7 mm-long nerve gap repaired with autograft (sural nerve) with/without light therapy (*n* = 12/group). Control: sham operated group without injury and repair; Control: sham operated group (*n* = 12)	4 months	Grasping test, muscle action potential measurements, histological analysis, morphometrical analysis
Nabian et al., 2011	Wistar	M	Right sciatic nerve and both median nerves (1 week later) were excised (*n* = 10); Both median nerves were excised, and the sciatic nerves were left intact (*n* = 10); Control: no surgical intervention (*n* = 10)	17 days	Sciatic functional index
Sinis et al., 2011b	Wistar	F	Floseal application to nerve stumps before nerve repair with end-to-end neurorrhaphy (*n* = 12); Electrocoagulation of nerve stumps before end-to-end neurorrhaphy (*n* = 12); Control: nerve repaired with end-to-end neurorrhaphy (*n* = 12)	3 months	Grasping test, muscle weight, histological analysis, morphometrical analysis
Chiono et al., 2009	Wistar	F	Repair of a 1.5 cm gap with PCL guides (2 cm long); Cross chest median/median nerve (5 cm PCL guides; 4.5 cm gap)	6 and 8 months, respectively	Grasping test, histological and immunohistochemical analysis
Nicolino et al., 2009	Wistar	F	10 mm-long nerve gap repaired with muscle-in-vein graft (*n* = 16); Nerve transection with no repair (*n* = 16)	5, 15, and 30 days	Histological analysis, expression analysis in muscle
Ronchi et al., 2009	Wistar	F	Crush injury (*n* = 14); Control (no injury) (*n* = 6)		Grasping test, histology, morphometrical analysis, TEM
Sinis et al., 2009	Wistar	F	Nerve repaired with end-to-end neurorrhaphy, with a wrapping by external jugular vein segment, filled with iron chelator DFO; Control: nerve repaired with end-to-end neurorrhaphy, with a wrapping by empty external jugular vein segment	3 months	Grasping test, muscle weight, histological analysis, morphometrical analysis, immunochemistry
Werdin et al., 2009	Wistar	F	Control group (*n* = 9); End-to-end nerve repair (*n* = 18) 2-cm autograft (*n* = 27)	12 weeks	Electrophysiological analysis
Audisio et al., 2008	Wistar	F	Nerve transection and repair with end-to-end neurorrhaphy (*n* = 10); Nerve transection and repair with end-to-side neurorrhaphy (distal stump sutured to the ulnar nerve) (*n* = 10); Control: healthy nerve (*n* = 10)	1, 2, and 3 weeks	Gene expression analysis
Ozalp and Masquelet, 2008	Wistar albino	M	5-mm gap repaired with silicon tube; after 5 weeks, the implant was removed and a nerve graft was anastomosed inside the neo-formed biological membrane (*n* = 10) 5-mm gap repaired with autograft (*n* = 10)	12 weeks	Grasping test
Geuna et al., 2007a	Wistar	F	Median nerve repaired with muscle-vein-combined conduit (*n* = 3/time point)	0 (2 h after preparation), 5, 15, and 30 days	IHC, Electron Microscopy, PCR
Geuna et al., 2007b	Wistar	F	End-to-side neurorrhaphy on the intact ulnar with a perineurial window; Tubulization by muscle-vein-combined guides Y-shaped muscle-vein-combined guides to repair both median and ulnar nerves	5 and 30 days	Grasping test, histological analysis (light, confocal and electron microscopy), PCR, muscle weight
Lee et al., 2007	Wistar	F	Controls (*n* = 4); End-to-end (median-to-median) (*n* = 4); End-to end (ulnar-to-ulnar) (*n* = 4); Median and ulnar nerve repaired with a 14-mm Y-shaped muscle-in-vein conduit (*n* = 4)	10 months	Grasping test, histological and morphometrical analysis
Papalia et al., 2007	Wistar	F	Median nerve repaired with end-to-side neurorrhaphy after epineurotomy on the radial nerve (*n* = 10)	30 weeks	Grasping test, histological, morphometrical and electrophysiological analysis
Sinis et al., 2007	Lewis	F	Control (*n* = 22); Autologous nerve graft (*n* = 22); Empty TMC/CL conduit (*n* = 16); TMC/CL conduit and SC (*n* = 22)	9 months	Grasping test, electrophysiological and histological analysis; muscle weight
Tos et al., 2007	Wistar	F	10-mm-long nerve defect repaired with muscle-in-vein conduit (15 mm long) using fresh muscle (*n* = 12); 10-mm-long nerve defected repaired with muscle-in-vein conduit (15 mm long) using freeze-thawed muscle (*n* = 12)	5 days, 1 month	Histological analysis, morphometrical analysis, gene expression analysis
Sinis et al., 2006	Lewis		Cross-chest (a gap of 40 mm was repaired with the two ulnar nerves) (*n* = 12); Control (healthy) (*n* = 12)	12 months	Grasping test, histological and morphological analysis
Bontioti et al., 2005	Wistar	F	End-to-side neurorraphy median/ulnar nerves to the musculocutaneous nerve (*n* = 11); End-to-side neurorraphy radial nerve to the musculocutaneous nerve (*n* = 11)	7 days and 6 months	Pawprints test, retrograde labelling, histological and morphometrical analysis, tetanic muscle force and muscle weight
Sinis et al., 2005	Lewis	F	Nerve defect of 2 cm repaired with resorbable hollow nerve conduit (*n* = 16); Nerve defect of 2 cm repaired with nerve conduit containing Schwann cells suspended in matrigel (*n* = 16); Nerve defect of 2 cm repaired with autograft (*n* = 22); Control: healthy nerve (*n* = 22)	9 months	Electrophysiology, grasping test, histological analysis, muscle weight
Gigo-Benato et al., 2004	Wistar	F	Complete nerve transection (15 mm gap) repaired with end-to-side neurorrhaphy on the ulnar nerve plus laser therapy (laser used: continuous, pulsed and a combination of the two) (*n* = 4/group). Nerve transection repaired with end-to-side neurorrhaphy on the ulnar nerve (*n* = 4); Controls: nerve transaction without repair (*n* = 4), healthy nerve (*n* = 5)	4 months	Grasping test, muscle weight, histological analysis, morphometrical analysis
Tos et al., 2004	Wistar	F	Controls (*n* = 5); Median and ulnar nerve repaired with a 14-mm Y-shaped muscle-in-vein conduit (*n* = 5)	8 months	Grasping test, histological and morphometrical analysis
Papalia et al., 2003a	Wistar	F	10 mm-long nerve defect repaired with end-to-side neurorrhaphy, through an epineurial window on the ulnar nerve (*n* = 20)	7 months	Grasping test, electrophysiology, muscle weight, histological analysis, morphometrical analysis
Papalia et al., 2003b	Wistar	F	End-to-side neurorrhaphy (*n* = 6)	16 weeks	Grasping test
Accioli De Vaconcellos et al., 1999	Sprague-Dawley	F	Control group (*n* = 8); Fresh autograft 20 mm long (*n* = 12); Frozen acellular autograft 20 mm long (*n* = 12); Fresh xenograft 20 mm long (*n* = 12); Frozen acellular xenograft 20 mm long (*n* = 12)	3, 6, 9, and 12 months	Grasping test, Retrograde labelling of neurons, Histological and histochemistry studies
Bertelli and Mira, 1995	Sprague-Dawley	F	Median and ulnar nerve bilateral dissection (*n* = 15); Left median nerve crush (*n* = 10); Left median nerve crush and right median nerve transection (*n* = 10); Left median nerve transection (*n* = 10); Left median and ulnar nerve transection (*n* = 10)	14 days, 3, 4, and 5 weeks	Grasping test, muscle weight

**Table 3:** Rabbit model.

References	Strain	Sex	Type of injury/gap	Follow up	Analysis
Sun et al., 2012	New Zealand rabbits	M/F	*In situ* anastomosis of the median nerves was made in parallel to the surrounding elbow veins, the transplanted epineurium and the adventitia were sutured with nerve anastomosis line (*n* = 30). Gap: 3 cm *In situ* anastomosis (control group); (*n* = 30). Gap: 3 cm	1, 2, 4, 8, and 12 weeks	Electrophysiological testing, and histopathology observation, TEM
Yin et al., 2011	New Zealand rabbits	–	Groups 2 and 3: Proximal median/ulnar nerve segment was served as father nerve to repair the distal nerve stump (Dor–Dor) (*n* = 6/group) Group 4: Serving as a donor nerve, the proximal 1/2 median nerve was fixed to the distal stumps of 1/2 median and ulnar nerve simultaneously, using biodegradable chitin conduits with a gap of 1 mm. (1/2 Dor - 1/2 Dor + Rec) (*n* = 6) Group 1: Control group (*n* = 6)	4 months	Electrophysiology, histology and morphometry
Kim et al., 2011	New Zealand rabbits	M	The ulnar nerve was transected and the distal end sutured to the median nerve 3 cm above the elbow (*n* = 30); The ulnar nerve was transected and the distal end sutured to the median 3 cm below the elbow joint (*n* = 30)	1, 2, 3, 4, 5, and 6 weeks	Morphometric analysis and immunohistochemistry.
Wang et al., 2009	New Zealand rabbits	–	Proximal nerve segment as donor nerve (*n* = 6) Intermediate nerve segment as donor nerve (*n* = 6); Distal nerve segment as donor nerve (*n* = 6); Right side nerves as control	3 months	Electrophysiology, histology and stereology; muscle weights
Zhang et al., 2006	New Zealand rabbits	–	End-to-side nerve coaptation performed immediately (*n* = 12); End-to-side nerve coaptation performed after nerve degeneration (*n* = 12); Control (*n* = 12)	3 and 6 months	Electrophysiology, Histomorphometry, Muscle weight
Baoguo et al., 2004	Japanese white rabbits	–	Study 1: the median nerve was elongated for 10 days (*n* = 6), for 15 days (*n* = 7) and for 20 days (*n* = 6). Study 2: Right arm: the proximal segment of an injured median nerve was elongated for 10 days (*n* = 10) and for 15 days (*n* = 10). Left arm: a 10- for 15-mm segment of the median nerve was removed, and a 10- for 15-mm segment, respectively, of the tibial nerve was grafted in its place.	4 months	Electrophysiology, Histomorphometry
Ruch et al., 2004	New Zealand rabbits	M	Group 1: nerve repaired with a tension-free medial antebrachial cutaneous graft (*n* = 11); Group 2: end-to-end repair without distraction (*n* = 13); Group 3: end-to-end repair with gradual distraction) (*n* = 12)	3 and 6 months	Electrophysiology, Histomorphometry, Muscle weight
Gui et al., 1997	–	–	The injured median nerve regenerated through degenerative latissimus dorsi muscle; The injured median nerve regenerated through the brachial triceps muscle	7, 14, 28, 45, 60, and 180 days	Histology
Shibata et al., 1991	–	–	Median nerve immediately repaired with the contralateral ulnar nerve graft (gap: 3-cm); Median nerve repaired with the contralateral ulnar nerve graft (gap: 3-cm) after resection of scar and coaptation at the distal site done 10 weeks later.	24 and 62 weeks	Electrophysiology, Histomorphometry, Muscle weight
Kawai et al., 1990	–	–	Nerve repaired with vascularised median nerve graft; Nerve repaired with non-vascularised median nerve graft. Length of the grafts: 2, 4, or 6 cm.	8 and 24 weeks	Histomorphometry
de la Monte et al., 1988	–	–	*n* = 34 total; Axonal regeneration across allografts (fresh or predegenerated) or autograft in cyclosporin A-treated/non-treated animals	–	Histology

**Table 4:** Sheep model.

References	Strain	Sex	Type of injury/gap	Follow up	Analysis
Kettle et al., 2013	–	–	Median-to-ulnar nerve end-to-side neurorrhaphy (*n* = 12); Conventional method of nerve repair (*n* = 18); Control (*n* = 8)	12 months	Electrophysiology and histology; Physiology of the muscle
Forden et al., 2011	Suffolk ewes	–	Defect of 5 cm repaired with 7 cm of radial sensory nerve (*n* = 12); Control (*n* = 1)	6 and 9 months	Electrophysiology, histology, immunohistochemistry, and morphometric analyses
Jeans L.A. et al., 2007	–	–	Microsurgical epineurial repair using 10/0 polyamide (*n* = 12); CRG-wrap and 6/0 polyglactin (*n* = 12); CRG-wrap and fibrin glue (*n* = 12)	7 months	Measure of transcutaneous stimulated jitter (TSJ), maximum conduction velocity (CVmax), wet muscle mass and morphometric measurements.
Jeans L. et al., 2007	–	–	Epineurial suture repair using 9/0 polyamide; CRG-wrap secured by Tisseel glue; CRG-wrap secured by polycaprolactone glue; Wrap secured by suturing (6/0 polyamide)	7 months	Electromyography, nerve conduction studies, wet muscle mass measurements, and morphometry
Kelleher et al., 2006a	–	–	Entubulation within a biodegradable glass tube (CRG tubes) (*n* = 6); Gap: 4/5 cm; Small magnets were applied to the sides of the biodegradable glass tube before the median nerve was repaired (*n* = 6); Gap: 4/5 cm Control (*n* = 6);	10 months	Morphometry electrophysiology and isometric tension assessment
Kelleher et al., 2006b	–	–	Median nerve repaired using an epineurial suture technique. CNTF was supplied into the CSF at the level of C6 by an implanted osmotic pump (*n* = 5). Median nerve repaired using an epineurial suture technique. Physiological saline was placed in the osmotic pump (*n* = 5); Control (*n* = 5);	6 months	Electrophysiological, morphometric and isometric tension experiments; muscle mass.
Matsuyama et al., 2000	–	–	Autograft (right side) and allograft (left side); immunosuppression with Cyclosporine A; 5-cm gap repaired with two cables of the radial sensory nerve (8-cm) (*n* = 4); Autograft (right side) and allograft (left side); (control *n* = 4)	between 35 and 47 days	Histology, Morphometry
Fullarton et al., 1998	Scottish black-faced sheep	F	Nerve immediately repaired with freeze-thawed muscle autografts (*n* = 5); gap 3 cm; Nerve repaired 30 days after injury with freeze-thawed muscle autografts (*n* = 5); gap 3 cm.	6 months	Electrophysiology and morphometry. Blood flow
Glasby et al., 1998	Scottish, black-faced sheep	F	Nerve immediately repaired with freeze-thawed muscle autografts (*n* = 5); gap 3 cm; Nerve repaired 4 weeks after injury with freeze-thawed muscle autografts (*n* = 5); gap 3 cm.	6 months	Electrophysiology and morphometry. Blood flow
Lawson and Glasby, 1998	Scottish, black-faced sheep	F	Nerve repaired with fascicular cable graft (*n* = 5); Nerve repaired with freeze-thawed muscle grafts (*n* = 5)	6 months	Nerve blood flow, nerve conduction velocity and morphological indices
Glasby et al., 1998	Scottish, black-faced sheep	F	Nerve immediately repaired with freeze-thawed muscle autografts (*n* = 6); gap 3 cm; Nerve repaired 30 days after injury with freeze-thawed muscle autografts (*n* = 6); gap 3 cm.	6 months	Electrophysiology and morphometry. Blood flow
Strasberg et al., 1996	–	–	Fresh nerve autograft (*n* = 5); Fresh nerve allograft (*n* = 5); Cold-preserved nerve (*n* = 5); Cold-preserved nerve allograft (*n* = 5)	6 and 10 months	Histological, morphometric, and electrophysiologic analyses.
Lawson and Glasby, 1995	Scottish, black-faced sheep	F	Nerve immediately repaired with freeze-thawed muscle autografts (*n* = 5); gap 3 cm; Nerve repaired 30 days after injury with freeze-thawed muscle autografts (*n* = 5); gap 3 cm.	6 months	Electrophysiology and morphometry. Blood flow

**Table 5:** Monkey model.

References	Strain	Sex	Type of injury/gap	Follow up	Analysis
Pace et al., 2014	Macaca fascicularis monkeys	F	Nerve repaired with bovine collagen I nerve conduit (NeuraGen) filled with keratin hydrogel (*n* = 8) (gap: 1 cm); Nerve repaired with bovine collagen I nerve conduit (NeuraGen) filled with sterile saline (*n* = 6) (gap: 1 cm)	12 months	Electrophysiology, nerve histology and morphometry, muscle histology and morphometry, antibody titer
Hu et al., 2013	Rhesus monkeys	–	Nerve defect (50 mm) repaired with: Autograft (*n* = 3); Chitosan/PLGA scaffold, followed by injection of autologous MSCs (*n* = 3); Chitosan/PLGA scaffold alone (*n* = 3); Nerve defect left untreated (control) (*n* = 3)	12 months	Locomotive activity observation, electrophysiological assessments, FG retrograde tracing tests, histological and morphometric analyses, blood test and histopathological examination
Hara et al., 2012	Macaca fascicularis	–	20-mm-long-segment was resected and repaired with: Lengthening of both nerve stumps (*n* = 3); Autograft with the sural nerve (*n* = 3);	16 weeks	Electrophysiological, histological, and functional recovery
Zhang et al., 2009	Rhesus monkeys	M	Right sides: small gap (2 mm) repaired with chitin conduit (length 10 mm); Left sides: traditional epineurium suture (*n* = 8)	6 months	Histology
Krarup et al., 2002	Macaca fascicularis	M	Nerve gap distances of 5, 20, or 50 mm were repaired with nerve grafts or collagen-based nerve guide tubes (total of 46 median nerve lesion). Control: direct repair	3–4 years	Electrophysiology
Florence et al., 2001	Macaca radiata	–	Median nerve was cut and sutured prenatally (*n* = 1); sensory enrichment of the nerve-injured hand; Median nerve was cut and sutured after birth (*n* = 5); 4 animals received sensory enrichment of the nerve-injured hand	3.5 months	Electrophysiological mapping studies (3b somatosensory cortex)
Florence et al., 1996	Macaque monkeys (immature)	–	Median nerve was cut and sutured prenatally (*n* = 2); Median nerve was cut and sutured after birth (*n* = 2)	10–18 months of age	Retrograde labelling to study the dorsal horn and cuneate nucleus; Electrophysiological mapping studies (3b somatosensory cortex)
Archibald et al., 1995	Macaca fascicularis	–	Autograft (gap 5 mm) in one side; In the contralateral wrist, the 5 mm nerve gap was bridged with a collagen nerve guide (*n* = 4); Direct suture (positive controls) (*n* = 4); Nerve gaps of mm bridged by polylactate nerve guides (*n* = 1). After 630–679 d the nerve guide was removed and the resulting gap of 15 mm was bridged by a collagen nerve guide.	Average of 1,342 d after surgery	Electrophysiology, motor conduction studies, sensory conduction studies, responses evoked by tactile stimulation, morphometric analyses.
Florence et al., 1994	Macaque monkeys	–	Median nerve was cut and sutured (*n* = 3)	7–13 months	Retrograde labelling to study the dorsal horn and cuneate nucleus; Electrophysiology to study the 3b of somatosensory cortex
Tountas et al., 1993	–	–	Median nerve repaired by microsurgical suture or tubulization with a non-woven, bioabsorbable, polyglycolic acid device (*n* tot = 30)	6 and 12 months	Electrophysiology and histology
Archibald et al., 1991	Macaca fascicularis	M	Nerve transected and repaired with: 4 mm nerve autograft (*n* = 3); Collagen-based nerve guide conduit (gap 4 mm) (*n* = 3)	760 days	Electrophysiology
Badalamente et al., 1989	Capuchin monkeys (*C. apella*)	-	Epineural repair (nerve stumps and thenar muscles were first bathed/injected with leupeptin) (*n* = 5); Epineural repair (nerve stumps were bathed with saline solution) (*n* = 5)	6 and 8 weeks, 3, 6 months	Electrophysiology, histology
Wall et al., 1986	Aotus trivirgatus	–	Nerves reconnected with 10/0 epineural sutures (*n* = 5)	From 76 to 322 days	Neurophysiological recording (cortical areas 3b and 1)
Grabb, 1968	Rhesus monkeys	F	Nerves were cut and sutured primarily (4 h after nerve injury, *n* = 30), or secondarily (about 3 weeks after injury, *n* = 30).	9 months	Electromyographic examination

In the following paragraph specificities of the different models listed in the tables will be reviewed in more detail.

### Mouse Model

Since most of the available transgenic animal models are mice, they are often used for studying the role of specific genes in the peripheral nerve regeneration process. For this purpose, genes of interest are knocked-out, mutated or over-expressed. Moreover, mice – especially wild type strains – are economical in their keeping, simple to handle and to care for and can therefore be studied in large groups. Mice nerves can be subjected to different types of injury and repair and analysed with all kinds of functional, morphological and biomolecular assays. The most simple crush injury can be easily used and standardised. On the other hand, peripheral nerves in mice are rather small and experimental *in vivo* work employing more complex surgeries on them, such as end-to-end repair, requires advanced microsurgical skills. These skills are provided by many clinical researchers from disciplines routinely performing nerve surgeries but are less prevalent in the basic researcher community. Furthermore, when it comes to studies evaluating new developments for bio-artificial nerve guides, the much smaller diameter of mouse peripheral nerves is not fitting the larger one of nerve guides that are primarily commonly designed to fit human digital nerves.

### Rat Model

Among rodents, rats are the most commonly used *in vivo* model in peripheral nerve regeneration research. This relates mainly to the dimension of their nerves. Surgery on rat peripheral nerves requires less microsurgical skills than needed for mouse models and rat limbs and nerves are long enough to allow 1.5–2 cm gap repair to be studied at least in the sciatic nerve. Moreover, rats can be investigated in large groups because their keeping is economical, and they are mostly simple to handle and to care for. Transgenic rat models are, however, less available than mouse transgenic models and also the availability of rat specific antibodies for molecular and histological examination is rather limited. But due to their prevalent employment, functional tests for evaluating motor or sensory recovery in rats are more standardised (Navarro, 2016) and comparable among different research groups. Rat nerves can be subjected to different types of injury and repair and analysed in most comprehensive functional, morphological and biomolecular assays (Ronchi et al., 2009; Navarro, 2016; Ronchi et al., 2016). Different rat strains can be utilised, for which varying willingness to enrol in specific functional tests is reported (Nikkhah et al., 1998; Galtrey and Fawcett, 2007), but so no comparative studies investigating differences in their ability to regenerate have been published.

When using rodents (mice and rats) as animal models for peripheral nerve regeneration, researchers must finally be aware of the following immanent differences to mankind: (1) gaps that can be produced are shorter than those commonly found in human nerve lesions; (2) axonal regeneration rate is faster than in humans; (3) recovery is often complete, while in humans it is often incomplete (Kaplan et al., 2015).

### Rabbit Model

In peripheral nerve regeneration research, rabbits offer the possibility to study regeneration across gap lengths of up to 6 cm. Rabbits are, however, expensive to purchase and maintain, and difficult to care for. Rabbits are more delicate and less resilient than rats and mice, and their occurrence as pet animals probably creates ethical problems for animal care takers and researchers. Also, there are almost no valid functional assays that can be applied in rabbit models, besides electrodiagnostic evaluation. Finally, very few specific antibodies are available to be used on rabbit tissue samples, so that conclusions on the regeneration outcome can mainly only be based on nerve morphometry studies.

### Sheep Model

The sheep as an animal model is useful when nerve regeneration across very long gaps should be evaluated. An ethical advantage of this animal is provided by the fact that a median nerve transection injury does not result in serious impairment of the limb usage ability and functional read-outs have been described in the recent years. To establish the model and to provide adequate housing conditions is, however, a considerable challenge, and research in this model will often only be realised through collaborative work.

### Monkey Model

Although non-human primates could be useful to test safety and efficacy of synthetic nerve conduits – because of the similarity of non-human primates with human beings – their use is considerably limited for ethical reasons. Monkeys are considered as animal models mainly to study neuronal plasticity occurring in the brain following peripheral nerve injury and repair. Again, studies in this model will mainly be subject of collaborative work and not appropriate for early stage pre-clinical research.

### Other Animals

Other large animals can be used as models to study median nerve injury and regeneration, such as pigs (Ochoa and Marotte, 1973; Marotte, 1974), dogs (Lee et al., 1999), and cats (Murray et al., 1997), but their use is limited for three main reasons: (1) animal care for large species is considerably expensive; (2) for some species the possibilities for functional testing are limited or require complex training, therefore, nerve regeneration may only be assessed with nerve morphometry; (3) dogs and cats are domestic animals, and their use in research is more restricted for ethical reasons.

## Methodological Considerations – Approaches for the Treatment of Nerve Injuries

The following criteria should be considered for selecting the most suitable animal model for pre-clinical research on peripheral nerve regeneration: (1) costs to purchase and house the animals; (2) ease of handling, such as tolerance to captivity; (3) tolerance for surgery and eventual repetitive anaesthesia, as well as resistance to infection; (4) compliance with national policies and with ethical principles; (5) inter-animal uniformity, life span of the species, biological information and available tools; (6) overall experimental plan (Angius et al., 2012). With regard to the latter, especially when several time-points are to be analysed, rodents represent the best choice. On the contrary, rodent life span is short, therefore for long experiments (>1–1.5 years), it becomes necessary to use lager animals as model organism.

In the last years, several approaches have been developed and tested in pre-clinical animal models to improve peripheral nerve regeneration. All these approaches can be applied on different nerves and are not median-nerve specific. In this paragraph we will present an overview of different techniques, including the use of different conduits to guide regenerating axons, the use of stem cell transplantation, the application of physical therapies and optogenetics, all of which have been demonstrating variable positive effects on nerve regeneration irrespectively of the model they were investigated in.

### Nerve Guidance Conduits

The major disadvantage of the autologous nerve graft technique is the remarkable sensitivity loss (mainly sural nerve grafts are harvested for this) and the limited availability of donor tissue (Ray and Mackinnon, 2010). Reconstruction of a nerve with artificial or non-artificial nerve conduit grafts is particularly indicated in case of extensive nerve tissue loss. Nerve guidance conduits made of several materials, artificial or of natural origin, are available; most common and FDA approved for clinical use are conduits made of collagen, chitosan, or poly (DL-lactide-ε-caprolactone) (Kornfeld et al., 2018). All devices currently on the market and FDA-approved have proven good support for the promotion of peripheral nerve regeneration in pre-clinical models. Good clinical results, however, are obtained only for lesions with a substance loss inferior to 3 cm in length, while severe and enlarged injuries remain a critical condition (Kaplan et al., 2015). For this reason, research in the field of novel nerve conduit functionalisation strategies is still highly vivid.

### Application of Stem Cells and Their Secretome

In regenerative medicine and tissue engineering, the use of Stem Cells and their secretome is fast expanding with the aim to develop innovative therapeutic strategies for the treatment of peripheral nerve injuries (Caplan, 2015; Caseiro et al., 2016; Busuttil et al., 2017; Sayad-Fathi et al., 2019). In particular, Mesenchymal Stem Cells (MSCs) present relevant key features: they can be easily expanded, they can differentiate into different cell types, they are immune-privileged and immune-modulatory, they show preferential homing to injured sites (Frausin et al., 2015; Sullivan et al., 2016; Jiang et al., 2017). Moreover, the MSC secretome contains trophic mediators (Meirelles Lda et al., 2009; Fu et al., 2017), modulating the function of several tissues, including the skeletal muscle (Pereira et al., 2014) and the peripheral nervous system (Lopatina et al., 2011; Gartner et al., 2012, 2014).

The most widely source of MSCs for therapeutic purposes is the bone marrow; as good alternative other sources are: the umbilical cord blood, the stromal tissue of the umbilical cord, the dental pulp, the adipose tissue (Jin et al., 2013).

### Transgenic Models to Promote Peripheral Nerve Regeneration

To study the biology of peripheral nerve regeneration, different transgenic models can be used (Magill et al., 2008). Most of the available transgenic animals are mice and they represent a powerful tool to study the influence of over-expression or depletion or mutation of a specific gene in a specific cell type, using inducible systems, but it must be kept in mind that mice are difficult subjects for microsurgical models due to the small size of their nerves, as discussed above.

To investigate the function of specific genes in nerve regeneration discriminating between motor and sensitive neurons, transgenic mice over-expressing the gene of interest in postnatal motoneurons or dorsal root ganglion neurons can be obtained using Thy1 or NSE (neuron specific enolase) promoters (Michailov et al., 2004; Gomez-Sanchez et al., 2009; Velanac et al., 2012).

To obtain tissue specific expression or depletion of specific proteins, the inducible cre-lox system can be applied: transgenic mice driving motor neuron specific expression of cre recombinase with the promoter of Mnx1 (motor neuron and pancreas homeobox 1) gene can be used to specifically in/activate the expression of floxed genes in motor neurons, while specific in/activation in Schwann cells can be obtained using the promoter of Mpz (myelin protein zero) gene (La Marca et al., 2011).

Transgenic animals can also be developed as advanced experimental models to study genetic diseases giving rise to peripheral neuropathies (Hoke, 2012; Juneja et al., 2018), and they can be studied to investigate their ability to regenerate injured peripheral nerves.

Finally, the expression of neurotrophic factors or other potentially therapeutic proteins in Schwann cells or in neurons can be obtained through the use of different viral vectors (Tannemaat et al., 2008).

### Physical Therapies

The efficacy of brief Electric Stimulation (ES) of the proximal stump of an injured nerve in promoting nerve regeneration in animal models has been verified in several independent studies reviewed by Gordon (2016) and Gordon and English (2016). In particular, a 14 days period of ES was chosen (Al-Majed et al., 2000) to accelerate the regenerative process and the effect was dramatic: preferential motor reinnervation of motor pathways was evident at 21 days rather than at 42 days, and, importantly, all of the motoneurons had regenerated into the motor nerve branch.

Another interesting study was aiming at evaluating the value of electromagnetic stimulation for the neural regenerative process of the rat median nerve after transection and end-to-end repair (Beck-Broichsitter et al., 2014b). From the 1st day after surgery a pulsed magnetic therapy was daily applied in the experimental group. Magnetic stimulation was positively influencing the functional regeneration in terms of grasping force and reduced muscular atrophy.

### Optogenetics

Axonal regeneration and functional recovery are enhanced by activity-related therapies, such as exercise and electrical stimulation (Gordon, 2016). Unlike electrical stimulation, optogenetics allows to selectively activate or inactivate specific neurons: for example, selective expression of the light-sensitive cation channel channelrhodopsin-2 (ChR2), that is maximally activated by blue light, can be used to depolarise neurons, thereby driving action potentials. Conversely, selective expression of the light-sensitive inward chloride pump halorhodopsin (Halo), that is maximally activated by amber light, can be used to obtain neuron hyperpolarisation, thereby inhibiting action potentials (Montgomery et al., 2016). Optically induced neuronal activity has been shown to be sufficient to promote functional motor axon regeneration *in vivo* (Ward et al., 2016). Moreover, through the selective expression of opsins in sensory neurons or motoneurons, it was possible to investigate the effect of system-specific neuronal activation on axonal regeneration, thus demonstrating that acute activation is sufficient to enhance regeneration of both motor and sensory axons (Ward et al., 2018).

Until now, in the peripheral nervous system, optogenetics has been applied mainly to sciatic nerves in transgenic mice expressing different opsins, but given its therapeutic potential it will be certainly applied also to median nerves and in other animal models, by injection of optogenetic constructs to transduce opsin expression in peripheral nerves in the future.

### Immunomodulation

The early inflammatory reactions undergoing in the course of Wallerian Degeneration of the distal nerve, comprise the activation of the complement system, arachidonic acid metabolites, and inflammatory mediators involved in myelin fragmentation and activation of repair Schwann cells. Fine-tuned upregulation of the cytokine/chemokine network by repair Schwann cells activates resident and hematogenous macrophages to complete the clearance of axonal and myelin debris and stimulate regrowth of axonal sprouts (Yona and Jung, 2010; Cortez-Retamozo et al., 2012; Dubovy et al., 2013; Jessen and Mirsky, 2016). An innovative approach in the field of peripheral nerve regeneration is exploiting the endogenous capacity of the body to repair itself through immune cells. In very promising studies, starting from the known different pro-inflammatory and pro-regenerative macrophage phenotypes, they were modulated through their response to different IFN-γ or IL-4 cytokines and studied in their ability to influence nerve regeneration in a critically sized, 15 mm rat sciatic nerve gap. The results of this research have shown that the administration of IL-4 at the injury site increased the pro-regenerative effect and therefore that the regenerative outcomes appeared to be influenced not only by the macrophage presence, but by their specific phenotype at the site of injury (Mokarram et al., 2012). A similar approach was conducted later by the same authors, through early stage administration of fractalkine, a chemokine able to control the phenotype in monocyte recruitment and to increase the regenerative potential. The pharmaceutical approach was evaluated from a morphological and functional point of view (Mokarram et al., 2017).

## Methodological Considerations – Techniques to Investigate Peripheral Nerve Regeneration

A number of different techniques have been developed to investigate the degree and the accuracy of nerve regeneration. While functional tests must be nerve-specific, all other methods can be applied to all types of peripheral nerves. In this paragraph we describe in detail different functional tests that are used to study the functional recovery of the median nerve and, in addition, we present an overview of other methods used for the investigation of nerve regeneration, including morphological and morphometrical analysis, gene expression analysis and fluorescent transgenic animal models.

### Functional Evaluation

Several functional tests are available for rodents, both rats and mice (Galtrey and Fawcett, 2007). Some have been designated *ad hoc* to evaluate functional recovery following median nerve repair (e.g., grasping test), others have been adapted from tests normally used following other lesion types (injuries to the spinal cord or sciatic nerves).

The tests described below refer to the rat, but most of them are adaptable also to mice.

• The *grasping test* is a simple method to assess the flexor function, first introduced by Bertelli and Mira (1995). The modified method (Papalia et al., 2003a) consists in presenting a small tower with only three bars forming a triangle on its top instead of a grid for grasping. With this modification the tendency to walk on the grid is avoided and the presence of a band put just below the three bars avoids that the rat employs the wrist flexion to hold the bars. This device is connected to a precision dynamometer. The animal is hold by its tail and allowed to grasp the grid. Then, the animal is pulled upward until it loses its grip. The balance records the maximum weight that the animal managed to hold before losing the grip.
• The *staircase test* is a functional test which assesses skilled forepaw reaching and grasping (Montoya et al., 1991). Two/three food pellets are placed on each step of two staircases located one on either side of a central platform. Both stairs are composed of seven steps. The animal is placed in a box and can only reach the pellets from the left staircase with its left paw and those from the right staircase with its right paw. The rat can grasp, lift, and retrieve food pellets from the steps of the staircase. The number of pellets completely removed from the staircase box provides a quantifiable measure of the distance and efficiency of fine motor reaching skills. Rats need to be pre-trained in the staircase test before surgery, put on restrictive diet before testing and need to be accomodated again to the test conditions for some days before the following evaluation. Therefore, this test is more complex to be applied than the grasping test described above.
• The *walking track analysis* is used to evaluate forelimb motor recovery (Ozmen et al., 2002; Galtrey and Fawcett, 2007). The rat forepaw is dipped in an ink solution and the animal is allowed to walk down the track upon a strip of white or graph paper. The prints by the ink are left to dry and then analysed. Different parameters can be analysed (longest length and widest width of the paw impression, widest width between the second and third fingers, distance between homologous points of sequential paw impressions on a given side, perpendicular distance between the central portion of the paw impression and the direction of movement). Moreover, walking track analysis can be performed by 2D digital video motion analysis, which allows also to quantify the movement of the wrist and the metacarpophalangeal joint (Wang et al., 2008).
• The *Von Frey filament test* is used to evaluate mechanical allodynia (Galtrey and Fawcett, 2007). The animal is placed in a box on a wire mesh floor. Von Frey filaments of different bending forces are used to examine the mechanical threshold of the rat forepaws. The test starts with the smallest bending force and continues in increasing order. Each filament is inserted through the mesh and applied in the medial surface of the forepaw. To perform the test, the rat must be stationary and standing on the four paws. The first filament in the series that evoked withdrawal three times is regarded as the paw withdrawal threshold.
• The *Irvine, Beattie, and Bresnahan (IBB) scale test* (Irvine et al., 2014) is used for the assessment of fine control of the forelimb and digit movements. Spherical- and donut-shaped pieces of cereal are given to the rat. The forelimb behaviour (joint position, object support, wrist and digit movement, and grasping method) used while eating both cereal shapes is analysed. An IBB score is assigned using the 10-point (0–9) ordinal scale for each shape, and the highest score reflects the greatest amount of forelimb recovery.
• The *ladder rung walking test* is used to assess forelimb strength, stepping, placing, and co-ordination during skilled locomotion (Metz and Whishaw, 2002; Galtrey and Fawcett, 2007). The apparatus consists of two side walls with rungs inserted into the walls to create a ladder. The ladder is elevated and can also be inclined. The animal is conditioned to run on the ladder on several training sessions. Performance is scored (successful steps/total steps).
• The *Randall-Selitto test* is used to assess the nociceptive withdrawal threshold (Galtrey and Fawcett, 2007). The test consists in the application of an increasing mechanical force with the tip of an algesimeter on the medial portion of the forepaw until a withdrawal response results.
• For the *cold sensory test* an ice probe is made by freezing water in a 1.5-ml tube (Lindsey et al., 2000; Galtrey and Fawcett, 2007). When the rat is drinking from the water bottle, the ice probe is applied to the glabrous skin of the forepaw. The withdrawal latency is measured. At least 1 min between trials is needed to allow the skin to return to body temperature and prevent sensitisation. If the rat does not withdraw the paw after 10 s, the probe is removed.
• The *cold and hot plate test* is used to assess temperature sensation. The rat is placed in a Plexiglas chamber where the metal base temperature is 25°C. For cold plate testing, the temperature is rapidly lowered and the animal behaviour is observed for signs of pain-like behaviour (avoiding contact with the cold plate, suspension of the affected forelimb, licking of the paw, lack of grooming and exploration vocalisation, or freezing behaviour). Once pain-like behaviour is observed, the temperature is increased to a higher temperature. For hot plate testing, the temperature is raised and the behaviour is assessed as described above (Shaikh et al., 2016).
• The *CatWalk* automated quantitative gait analysis is a computer-assisted method that can simultaneously measure dynamic as well as static gait parameters, including duration of different phases of the step cycle and pressure applied during locomotion (Bozkurt et al., 2008). The animal is placed in the CatWalk walkway, which is comprised of a glass plate with two Plexiglas walls, a high-speed colour camera, and recording and analysis software. The animal walks voluntarily from one side of the glass plate to the other. Its footprints are captured. The intensity of the signal depends on the degree of paw floor contact and increases with pressure applied. The more pressure is exerted, the larger the total area of skin–floor contact and thus the brighter the pixel. An appropriate software visualises the prints and calculates statistics related to print dimensions and the time and distance relationships between footfalls (Chen et al., 2012a,b).
• *Electrophysiology* is often used in rat, while in the mouse model it is less used probably for the small size. The maximum amplitude and latency of evoked compound muscle action potentials recorded from the thenar muscles are usually evaluated (Werdin et al., 2009).

In conclusion, the list provided above clearly demonstrates that a large variety of tests can be used to evaluate the functional recovery after median nerve transection and repair in rodents.

From a translational point of view, tests should be selected in way to model as closely as possible the course of functional recovery as it is observed in human patients. Recently, the combination of electrodiagnostic evaluation, with the commonly used grasping test (reflex-based gross motor function) and the staircase test (skilled forelimb reaching) has been described to produce results with high translatability (Stossel et al., 2017).

A final comment needs to be put on the fact that different rat strains have been described to demonstrate different motivation degrees to participate in more complex tasks, like, e.g., the staircase test. Especially, Lewis rats have been described to be less motivated and also to eventually be less capable of learning how to perform more complex motor tasks (Nikkhah et al., 1998; Galtrey and Fawcett, 2007).

In the other animals, the functional recovery is assessed mainly by electrophysiology. Indeed, while in rats and mice the fingers do a fine movement quite similar to humans and their functionality can be assessed by different suitable and specific tests, the other animals can do gross movements only.

### Morphology and Morphometry (Stereology)

Regardless of the animal model used, nerve regeneration assessment must necessarily have an accurate morphological and morphometrical evaluation (Geuna and Herrera-Rincon, 2015). Among the techniques that allow this type of analysis, immuno-histochemistry offers the possibility to specifically identify the different structures of the regenerating nerve, such as Schwann cells, motor or sensory axons, blood vessels, and other cell types, including macrophages, fibroblast-like cells, perineurial cells, endothelial cells (Carriel et al., 2014b). Moreover, immunofluorescence or immunohistochemical techniques allow to accurately quantify the fraction area and the intensity of the expression of specific proteins which are correlated with regenerative processes. For example, the expression of markers such as Neurofilament in neurons or S-100 in Schwann cells are indicative of an excellent regenerative process, when these levels reach the values of the control nerves (Carriel et al., 2014a).

To quantify the number of sensitive and motor neurons which were able to regenerate axons across a nerve gap, the retrograde-labelling technique can be applied. A dye will be applied into the distal nerve and taken up by regenerated axons and retrogradely transported into the neuronal soma of sensory neurons in the dorsal root ganglia or motor-neurons in the ventral horn of the spinal cord (Hayashi et al., 2007; Kemp et al., 2017).

Together with functional assessment, quantitative estimation of regenerated nerve fibres is a key investigation tool in nerve regeneration research (Kanaya et al., 1996; Geuna et al., 2004). The toluidine blue staining of resin-embedded semithin sections allows to clearly identify most of the myelinated axons and their myelin organisation thanks to the post-fixation with OsO_4_ (Raimondo et al., 2009). Usually, morpho-quantitative analysis is performed on one randomly selected toluidine blue semi-thin transverse nerve section. The total cross-sectional area of the nerve is measured. Then, an adequate number of fields of interest (according to the size of the nerve) is randomly selected following a systematic random protocol and analysed (Raimondo et al., 2009). The parameters used as nerve regeneration indicators are myelinated fibre number and density, fibre and axon diameter, myelin thickness and *g*-*ratio* (axon-diameter/fibre-diameter) (Geuna, 2000, 2005).

In order to compare results obtained by different research groups, different potential sources of bias should be considered.

First of all, the strain, the gender and the age of experimental animals, that can affect the quantification outcome.

The second aspect that can influence the results of a stereological analysis is the level at which it is conducted and, obviously, the different investigation time points. The analysed parameters can vary significantly depending on the distance from the lesion point, especially in the early time points after injury, considering that a nerve can grow approximately 1 mm/day (Santos et al., 2007). Therefore, only quantitative data taken at the same location along the nerves can actually be compared (e.g., 5 mm distal to the lesion site). Obviously, also the time point analysed gives different results and should always be considered (with the same lesion type, a 3-month regenerated nerve will be different from a 6-month regenerated nerve). Finally, it must possibly be considered to analyse all branches of a nerve. The portion of the median nerve that is usually injured and repaired in experimental studies goes from the axillary region to the elbow. In this tract, the median nerve is unifascicular, but for more distal investigation sites the anatomy of the nerve needs to be considered. The median nerve gives off three palmar digital branches more distally, at the level of the carpal bones, that in turn bifurcate between the 1st, 2nd, and 3rd digit (Barton et al., 2016). Other nerves (i.e., the sciatic nerve) release branches in the tract that is commonly investigated. Therefore, if the morphological/morphometrical analysis requires the nerve cross section (for example to estimate the total number of nerve fibres), all branches must be analysed.

The third aspect is represented by the chosen method for measuring the selected size parameters (computerised or manual analysis). It is important to note how computers can certainly make quantitative morphology easier and faster (Williams and Rakic, 1988; Dolapchieva et al., 2000), but a comparison of the performance of automated cell detection revealed that a manual approach is still the most appropriate method for stereological counting (Schmitz et al., 2014).

### Gene Expression Analysis

Injured median nerve gene expression analysis can be carried out both at mRNA and protein level. To reduce the number of animals and comply with the 3R’ Principle (Replace, Reduce, and Refine) (Tannenbaum and Bennett, 2015) a good strategy is to extract from the same nerve sample both total RNA and proteins, using commercially available kits.

The first point that should be considered when analysing a nerve sample is that protein extraction involves all nerve components, neuronal axons and peripheral cells (Schwann cells, fibroblasts, macrophages, and so on). RNA extraction mostly encloses peripheral cells, because neuronal RNA is mainly localised in the cell bodies of sensory neurons (in the dorsal root ganglia) or motor neurons (in the ventral horn of the spinal cord), with only few mRNAs locally translated in the axon after nerve injury (Terenzio et al., 2018).

The second point that should be carefully considered is the portion of the injured nerve to be analysed and the time window for the analysis. Indeed, in the 1st days after injury, regenerating axons start to colonise the proximal portion of the repaired nerve, while in the distal portion axons are still undergoing Wallerian degeneration (Girouard et al., 2018). In the following days, regeneration occurs also in the distal stump. Therefore, gene expression analysis will give information about regeneration or Wallerian degeneration taking place according to the region and the time point analysed: for gene expression analysis, like previously discussed for morphology and morphometry, the region and the time point analysed must be the same for all samples and for comparison with other studies.

For mRNA analysis, quantitative real time PCR can be carried under paying attention to the housekeeping genes used for normalisation: indeed, it is really important to normalise data to genes whose expression is not affected by nerve injury (Vandesompele et al., 2002). To this aim, stable housekeeping genes suitable for gene expression analysis were identified (Gambarotta et al., 2014; Wang et al., 2017). To avoid amplification of contaminant genomic DNA, a good strategy is to design primers on different exons, possibly separated by introns larger than 1,000 bp, or straddling two contiguous exons. For protein analysis, western blots can be carried out using the stain-free technology, which is a good strategy, because protein expression is normalised to the total protein content, bypassing the problem of the choice of suitable housekeeping genes (Gurtler et al., 2013).

### Transgenic Models to Evaluate Peripheral Nerve Regeneration

A transgenic model that can be used for evaluating and monitoring peripheral nerve regeneration in mice and rats is Thy1-GFP, in which Thy1 promoter drives neuron specific expression of GFP, allowing imaging of nerve regeneration following nerve injuries (Porrero et al., 2010; Moore et al., 2012; Kemp et al., 2013).

## Conclusion

In this review we emphasised the use of the median nerve as pre-clinical experimental model to study nerve regeneration *in vivo*. Accordingly, in the last years the median nerve model is increasingly used due to different reasons, including a better animal well-being, and availability of different functional tests which, specifically in rodents, allow testing the digital fine movement, making the results obtained with this model more translatable into the clinic.

With regard to the animal choice, the rat definitely represents the easiest and more translatable model, especially for studies evaluating regeneration across short nerve gaps, while for large nerve gaps the use of other, larger, animals would be recommended. In case large animal facilities are not available, an alternative and very interesting approach might be the cross-chest median nerve transfer in the rat animal model (Sinis et al., 2006). Indeed, this method would allow the use of rodents, which are less expensive and easier to handle compared to large animals, but at the same time would allow studying nerve regeneration across long gaps (up to 40 mm).

## Author Contributions

GR, GG, SG, and KH-T organised the manuscript. GR, GG, and MM prepared all the tables. GR, GG, MM, FF, SG, and KH-T wrote different sections of the manuscript. All authors contributed to manuscript revision, and read and approved the submitted version.

## Conflict of Interest Statement

The authors declare that the research was conducted in the absence of any commercial or financial relationships that could be construed as a potential conflict of interest. The handling Editor is currently co-organizing a Research Topic with two of the authors KH-T and GG, and confirms the absence of any other collaboration.
